# Health, Quality of Life, and Economic Impacts of Home Care Vouchers for Middle-Income Adults With Disabilities

**DOI:** 10.1093/geroni/igaa057.256

**Published:** 2020-12-16

**Authors:** Joanne Spetz, Jacqueline Miller, Connie Kwong, Laura Wagner

**Affiliations:** University of California, San Francisco, San Francisco, California, United States

## Abstract

The Support at Home pilot program provided financial support for the purchase of home care services by middle-income adults with disabilities in San Francisco to support aging in place. Enrollees had income below the area median and made copayments based on household income. The mixed-methods evaluation of the program incorporated administrative records, surveys of clients and comparison group members, surveys of unpaid caregivers, surveys of paid care providers, and focus groups with clients and unpaid caregivers. Outcome measures included the Older People’s Quality of Life Questionnaire, Patient Health Questionnaire-2, an adapted Burden Scale for Family Caregivers, and self-reported falls, emergency department visits, and hospitalizations. Analyses included pre-post chi-squared and t-test comparisons between client and comparison groups and multivariate regressions. An economic analysis was conducted to learn whether changes in costs associated with reduced health care utilization were greater than the costs of the program. Results indicated statistically significant positive changes in client ratings of personal and financial stress, but not in the composite quality of life score. There were statistically significant reductions in attendance at medical appointments, falls, emergency department visits, and hospitalizations. Similar changes were not found in the comparison group. The focus group data supported the findings regarding personal and financial stress and indicated that clients and their caregivers perceived quality of life benefits. The economic analysis indicated substantial cost savings from the program due to reduced use of medical services. Due to its positive impacts, San Francisco has made Support at Home a permanent program.

